# Association of *KRAS* variants with survival and therapeutic outcomes in biliary tract cancers

**DOI:** 10.1016/j.esmoop.2025.105306

**Published:** 2025-06-10

**Authors:** K. Iida, Y. Matsui, Y. Urabe, T. Muramatsu, J. Matsuzaki, Y. Saito

**Affiliations:** 1 Division of Pharmacotherapeutics, Keio University Faculty of Pharmacy, Minato-ku, Tokyo; 2 Division of Gastroenterology and Hepatology, Department of Internal Medicine, Keio University School of Medicine, Shinjuku-ku, Tokyo, Japan

**Keywords:** *KRAS* variants, biliary tract cancer, intrahepatic cholangiocarcinoma, extrahepatic cholangiocarcinoma, gall-bladder cancer

## Abstract

**Background:**

Biliary tract cancer (BTC) remains a highly aggressive malignancy with limited treatment options and poor prognosis. To explore the association between *KRAS* variants and survival/therapeutic outcomes of patients with BTC, we analyzed genetic and clinical data obtained from BTC patients who underwent comprehensive genomic profiling (CGP) testing in Japan.

**Patients and methods:**

A total of 7773 patients with BTC who were registered in the Center for Cancer Genomics and Advanced Therapeutics (C-CAT) were included in this study. The main outcome was overall survival (OS) in months. For survival analysis, OS was measured from diagnosis to last follow-up or death, and for therapeutic outcomes, from treatment initiation to last follow-up or death.

**Results:**

The overall frequency of *KRAS* mutations in BTC was 23.4%. When classified according to tumor subtype, *KRAS* mutations were identified in 24.9% of patients with intrahepatic cholangiocarcinoma (IHC), in 32.2% of those with extrahepatic cholangiocarcinoma (EHC), and in 9.4% of those with gall-bladder cancer (GB). Among patients with *KRAS* mutations, G12D mutation was the most common in IHC (41.5%), EHC (35.9%), and GB (29.8%). In all BTC subtypes (IHC, EHC, and GB), patients with *KRAS* mutations had worse OS compared to those with wild-type *KRAS*. By *KRAS* variant, the G12D, G12V, and Q61H mutations were associated with poor OS in patients with IHC, while the G12D and G12V mutations were associated with poor OS in patients with EHC. Furthermore, in patients with unresectable BTC who received first-line treatment with regimens including gemcitabine, cisplatin, and durvalumab therapy, the G12D mutation was associated with poor OS across all regimens evaluated in this study.

**Conclusions:**

*KRAS* variants were significantly associated with poor prognosis and unfavorable therapeutic outcomes in BTC patients and may serve as potential prognostic and predictive factors.

## Highlights

• Using data from >7000 biliary tract cancers, we examined the link between *KRAS* variants and prognosis/treatment outcomes.
• In patients with biliary tract cancer, *KRAS* variants were significantly associated with poor prognosis.
• *KRAS* variants were associated with poor treatment outcomes in patients with biliary tract cancer.
• *KRAS* variants maybe BEredictive for survival
• *KRAS* variants may be potential prognostic and predictive markers for survival/treatment outcomes in biliary tract cancers.

## Introduction

Biliary tract cancer (BTC), a highly aggressive malignancy, comprises intrahepatic cholangiocarcinoma (IHC), extrahepatic cholangiocarcinoma (EHC), and gall-bladder cancer (GB). Despite recent advances in surgical treatment, drug therapy, and personalized medicine, the prognosis of BTC remains poor.^1^^,^^2^ The 5-year overall survival (OS) rate for advanced-stage BTC is <20%, and many patients are diagnosed at an inoperable stage.^3^, ^4^, ^5^

Gemcitabine plus cisplatin (GC) therapy is the standard first-line treatment of advanced BTC; however, the efficacy of GC therapy remains limited, and most patients eventually experience disease progression.^6^ Gemcitabine, cisplatin, and S-1 (GCS) therapy is emerging as one of the standard treatment options for advanced BTC in Japan.^7^ In addition, recent advances in cancer immunotherapy have raised hopes of improved outcomes for those with BTC. The combination of GC therapy with immune checkpoint inhibitors (ICIs) such as durvalumab (GCD therapy) and pembrolizumab has shown promise in clinical trials.^8^, ^9^, ^10^ The TOPAZ-1 trial demonstrated that GCD therapy prolongs OS significantly when compared with GC alone.^8^^,^^10^ These findings suggest that integrating ICIs into the treatment paradigm could provide new therapeutic options for BTC patients; however, as their additional benefits over GC therapy remain limited, identifying factors that predict the efficacy of ICIs is a crucial challenge.

One of the most common driver gene mutations in BTC is the *KRAS* mutation. As a key regulator of the RAS/MAPK signaling pathway, KRAS controls cell proliferation and differentiation; mutation in the gene results in persistent activation of this pathway, thereby driving tumor progression.^11^ Hotspot mutations in the *KRAS* gene are located at codons 12 (G12), 13 (G13), and 61 (Q61). Recent studies report that *KRAS* variants affect the prognosis of patients with colorectal cancer, lung cancer, and BTC.^12^, ^13^, ^14^, ^15^, ^16^, ^17^, ^18^ However, the detailed association between *KRAS* variants and OS in BTC patients with different tumor subtypes (IHC, EHC, or GB), as well as their therapeutic outcomes, remains unclear.

In this cohort study, we used data from a large-scale database provided by the Center for Cancer Genomics and Advanced Therapeutics (C-CAT) to comprehensively evaluate the association between *KRAS* variants and survival/therapeutic outcomes in patients with BTC. C-CAT, a national cancer genomic database in Japan, was established to advance precision oncology by integrating clinical and genomic data from cancer patients across multiple medical institutions. C-CAT serves as a central repository for comprehensive genomic profiling (CGP) data.^19^ As of December 2024, C-CAT has accumulated genomic and clinical data from >93 000 cancer patients, making it one of the largest cancer genome databases in Japan.^19^, ^20^, ^21^, ^22^ This comprehensive dataset allowed us to evaluate the association between *KRAS* variants and OS/treatment efficacy in patients with BTC, providing new insight into BTC biology and future therapeutic strategies.

## Materials and methods

### Clinical and genomic information

This retrospective cohort study utilized data from the C-CAT database (https://www.ncc.go.jp/en/c_cat/index.html). The C-CAT database comprises clinical and genomic data from cancer patients in Japan, all of whom have provided written informed consent to their use for research purposes. Genomic information is extracted from CGP data using NCC OncoPanel, FoundationOne CDx, FoundationOne Liquid CDx, Guardant360 CDx, and GenMineTOP. Clinical data include age, sex, cancer type, pathological diagnosis, clinical stages, performance status, history of smoking (cigarette smoking only) and alcohol consumption, and metastatic status. Moreover, the database contains details about treatment regimens, including the start and end dates, antitumor responses, and occurrence of severe adverse events during treatment. Genomic information includes the type of abnormal genes detected, the mutation type and its frequency and clinical significance, and microsatellite instability. We analyzed the genetic and clinical data of BTC patients who underwent CGP testing, almost all of whom were registered in the C-CAT database, between June 2019 and January 2025. The study was reviewed and approved by the institutional review board at Keio University (Tokyo, Japan), and by the C-CAT Data Utilization Review Board (CDU2023-039).

### Statistical analysis

Patient characteristics are expressed as the median and interquartile range (IQR; continuous variables), or as numbers and percentages (categorical variables). Between-group comparisons were made using one-way analysis of variance and Pearson’s *chi*-squared test.

Kaplan–Meier survival analyses were conducted to evaluate OS. For survival analysis, OS was measured from diagnosis to last follow-up or death, and for therapeutic outcomes, from treatment initiation to last follow-up or death. Comparisons between subgroups were carried out using the log-rank test. Multivariate analysis was carried out using the Cox proportional hazards regression model. Statistical significance was defined as *P* < 0.05, and all analyses were carried out using R version 4.3.3 and SPSS version 29.0.2.

## Results

### Frequency of KRAS variants in BTC patients

**Figure 1:**
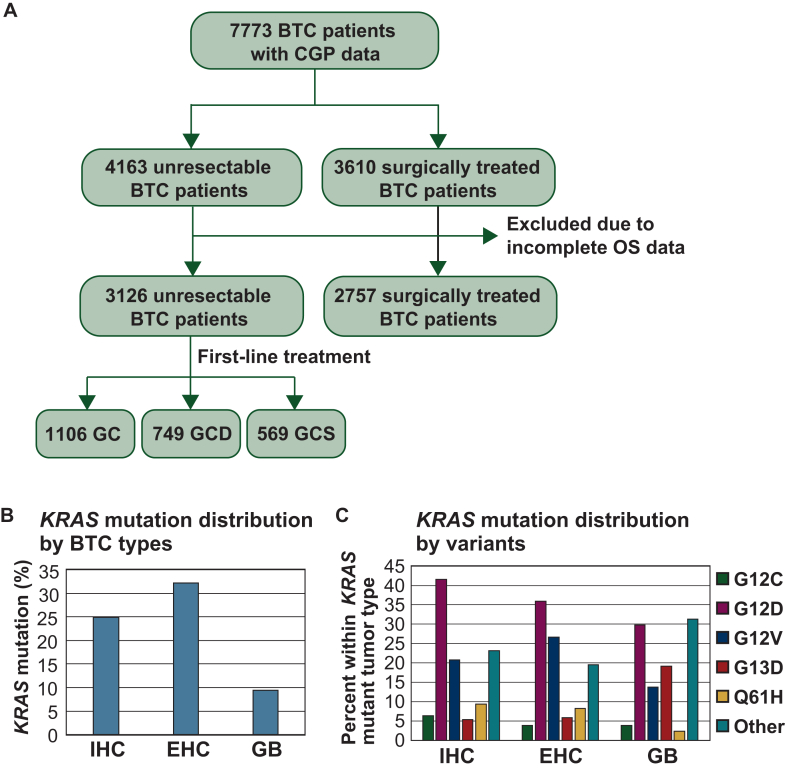
**Patients included in the study, and the frequency of *KRAS* variants.** (A) A total of 7773 BTC patients with CGP data in the C-CAT database were enrolled in the study. Among these, 3610 (46.4%) were classified as resectable BTC. The remaining 4163 cases (53.6%) were classified as unresectable BTC. After excluding cases with incomplete OS data, 3126 patients with unresectable BTC were available for OS analysis. Among them, those who received first-line treatment were categorized into the following groups: GC (*n* = 1106), GCD (*n* = 749), and GCS (*n* = 569). (B) Frequency of *KRAS* mutations in BTC patients according to tumor subtype. (C) Frequency of *KRAS* variants in BTC patients according to tumor subtype. BTC, biliary tract cancer; CGP, comprehensive genomic profiling; EHC, extrahepatic cholangiocarcinoma; GB, gall-bladder cancer; GC, gemcitabine + cisplatin; GCD, gemcitabine + cisplatin + durvalumab; GCS, gemcitabine + cisplatin + S-1; IHC, intrahepatic cholangiocarcinoma; OS, overall survival.

A total of 7773 BTC patients with CGP data in the C-CAT database were enrolled in the study (Figure 1A). Among these, 3610 (46.4%) were classified as resectable BTC, including patients who underwent neoadjuvant or adjuvant therapy, and those whose surgical specimens were used for CGP testing. The remaining 4163 cases (53.6%) were classified as unresectable BTC. After excluding cases with incomplete OS data, 3126 patients with unresectable BTC were available for OS analysis. Among them, those who received first-line treatment were further categorized into the following groups based on the treatment regimen: GC (*n* = 1106), GCD (*n* = 749), and GCS (*n* = 569) (Figure 1A). Supplementary Tables S1 and S2, available at https://doi.org/10.1016/j.esmoop.2025.105306 summarize patient characteristics according to tumor type and first-line treatment, respectively.

We examined the frequency of *KRAS* mutations in BTC (Figure 1B and Supplementary Table S1, available at https://doi.org/10.1016/j.esmoop.2025.105306). The overall frequency of *KRAS* mutations in BTC was 23.4% (*n* = 1378/5883). Stratification by tumor subtype revealed *KRAS* mutations in 24.9% (*n* = 554/2227) of patients with IHC, 32.2% (*n* = 513/1593) of those with EHC, and 9.4% (*n* = 131/1388) of those with GB (Figure 1B). Upon investigating *KRAS* mutation variants, G12D mutation was the most common in IHC (41.5%, *n* = 230/554), followed by EHC (35.9%, *n* = 184/513), and GB (29.8%, *n* = 39/131) (Figure 1C and Supplementary Table S1, available at https://doi.org/10.1016/j.esmoop.2025.105306). In both IHC and EHC, the second most common *KRAS* mutation was G12V (20.8%, *n* = 115/554 and 26.7%, *n* = 137/513), and the third was Q61H (9.4%, *n* = 52/554 and 8.2%, *n* = 42/513). By contrast, in GB, the second most common *KRAS* mutation was G13D (19.1%, *n* = 25/131), and the third was G12V (13.7%, *n* = 18/131). Among *KRAS* mutations, G12C accounted for 6.3% (*n* = 35/554) in IHC, 3.9% (20/513) in EHC, and 3.8% (5/131) in GB (Figure 1C and Supplementary Table S1, available at https://doi.org/10.1016/j.esmoop.2025.105306).

### Association between KRAS variants and OS in BTC patients

**Figure 2:**
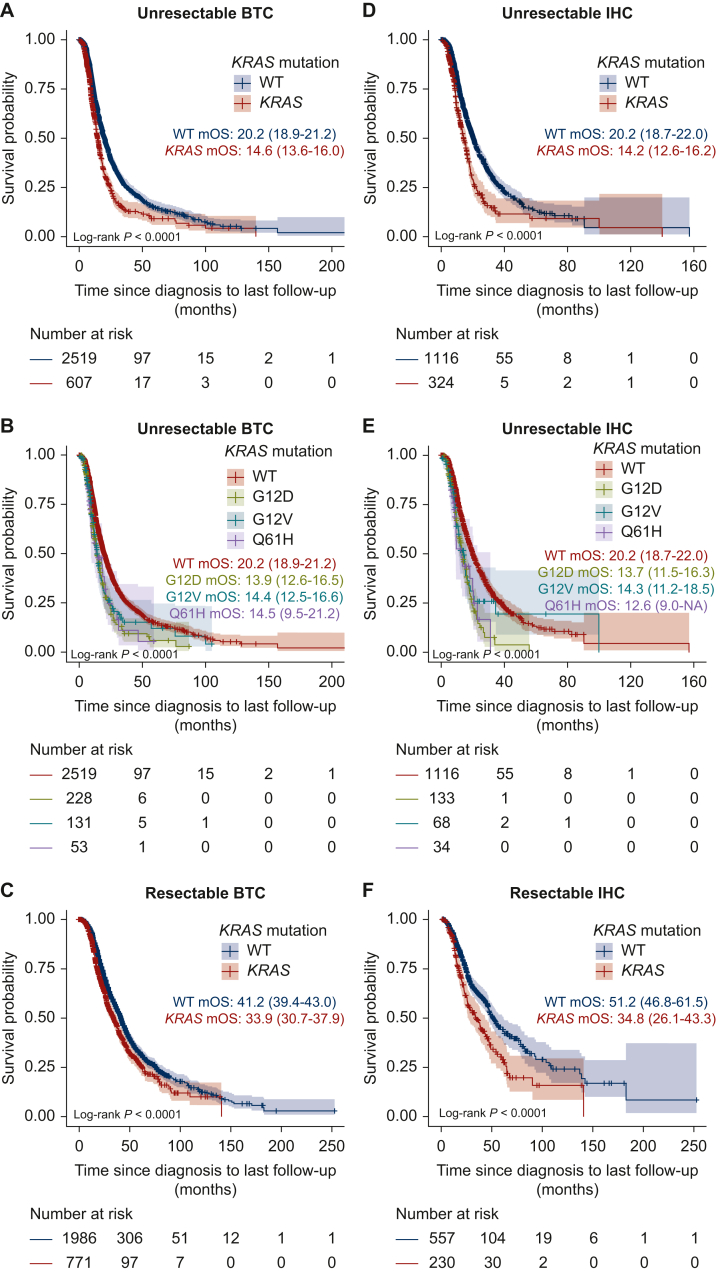
**Association between *KRAS* variants and overall survival (OS) of patients with biliary tract cancer (BTC) and intrahepatic cholangiocarcinoma (IHC).** (A-C) Kaplan–Meier curves for patients with unresectable and resectable BTC showing OS stratified according to *KRAS* variants. (D-F) Kaplan–Meier curves for patients with unresectable and resectable IHC showing OS stratified according to *KRAS* variants. OS was measured in months from diagnosis to last follow-up or death, and the median OS (95% CI) for each group is shown. BTC, biliary tract cancer; CI, confidence interval; IHC, intrahepatic cholangiocarcinoma; mOS, median overall survival; WT, wild-type.

Next, we investigated the association between *KRAS* mutation status and OS in BTC patients, and found that *KRAS* mutations were significantly associated with worse OS in both unresectable BTC [median OS: wild-type (WT), 20.2 months (95% CI 18.9-21.2 months); mutant *KRAS*, 14.6 months (95% CI 13.6-16.0 months); log-rank *P* < 0.0001; Figure 2A] and resectable BTC [median OS: WT, 41.2 months (95% CI 39.4-43.0 months); mutant *KRAS* 33.9 months (95% CI 30.7-37.9 months); log-rank *P* < 0.0001; Figure 2C].

**Figure 3:**
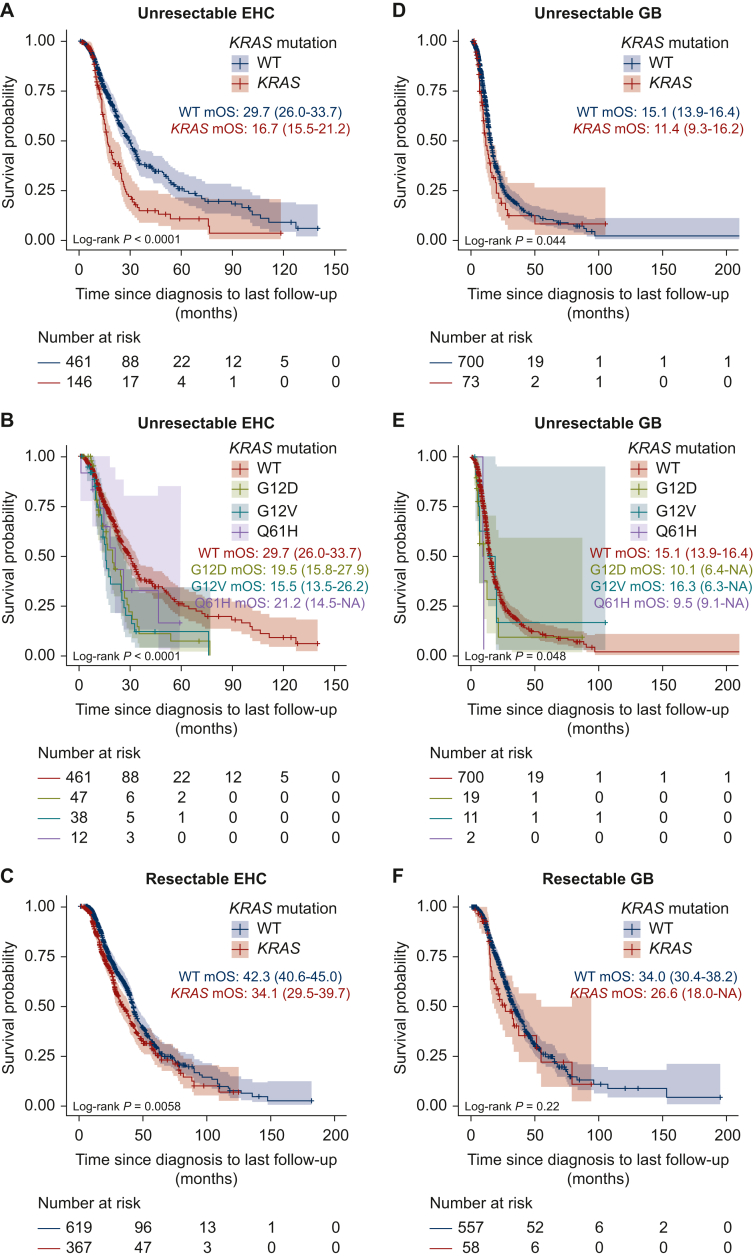
**Association between *KRAS* variants and overall survival (OS) of patients with extrahepatic cholangiocarcinoma (EHC) and gall-bladder cancer (GB).** (A-C) Kaplan–Meier curves for patients with unresectable and resectable EHC showing OS stratified by *KRAS* variants. (D-F) Kaplan–Meier curves for patients with unresectable and resectable GB showing OS stratified by *KRAS* variants. OS was measured in months from diagnosis to last follow-up or death, and the median OS (95% CI) for each group is shown. CI, confidence interval; EHC, extrahepatic cholangiocarcinoma; GB, gall-bladder cancer; mOS, median overall survival; WT, wild-type.

Subgroup analyses revealed that *KRAS* mutations were associated with worse OS in both unresectable IHC [median OS: WT, 20.2 months (95% CI 18.7-22.0 months); mutant *KRAS*, 14.2 months (95% CI 12.6-16.2 months); log-rank *P* < 0.0001; Figure 2D] and resectable IHC [median OS: WT, 51.2 months (95% CI 46.8-61.5 months); mutant *KRAS*, 34.8 months (95% CI 26.1-43.3 months); log-rank *P* < 0.0001; Figure 2F]. *KRAS* mutations were also associated with worse OS in both unresectable EHC [median OS: WT, 29.7 months (95% CI 26.0-33.7 months); mutant *KRAS*, 16.7 months (95% CI 15.5-21.2 months); log-rank *P* < 0.0001; Figure 3A] and resectable EHC [median OS: WT, 42.3 months (95% CI 40.6-45.0 months); mutant *KRAS*, 34.1 months (95% CI 29.5-39.7 months); log-rank *P* = 0.0058; Figure 3C]. In contrast, in GB patients, *KRAS* mutations were associated with poor prognosis in unresectable cases [median OS: WT, 15.1 months (95% CI 13.9-16.4 months); mutant *KRAS*, 11.4 months (95% CI 9.3-16.2 months); log-rank *P* = 0.044; Figure 3D] but showed no significant association in resectable cases [median OS: WT, 34.0 months (95% CI 30.4-38.2 months); mutant *KRAS*, 26.6 months (95% CI 18.0 months to NA); log-rank *P* = 0.22; Figure 3F].

We then examined the association between the three most frequent *KRAS* mutations (G12D, G12V, and Q61H) and OS in patients with unresectable BTC. *KRAS* variants were associated with OS [median OS: WT, 20.2 months (95% CI 18.9-21.2 months); G12D, 13.9 months (95% CI 12.6-16.5 months); G12V, 14.4 months (95% CI 12.5-16.6 months); Q61H, 14.5 months (95% CI 9.5-21.2 months); log-rank *P* < 0.0001; Figure 2B]. Similarly, *KRAS* variants were associated with OS in patients with unresectable IHC [median OS: WT, 20.2 months (95% CI 18.7-22.0 months); G12D, 13.7 months (95% CI 11.5-16.3 months); G12V, 14.3 months (95% CI 11.2-18.5 months); Q61H, 12.6 months (95% CI 9.0 months to NA); log-rank *P* < 0.0001; Figure 2E]. *KRAS* variants were also associated with OS of patients with unresectable EHC [median OS: WT, 29.7 months (95% CI 26.0-33.7 months); G12D, 19.5 months (95% CI 15.8-27.9 months); G12V, 15.5 months (95% CI 13.5-26.2 months); Q61H, 21.2 months (95% CI 14.5 months to NA); log-rank *P* < 0.0001; Figure 3B] and unresectable GB [median OS: WT, 15.1 months (95% CI 13.9-16.4 months); G12D, 10.1 months (95% CI 6.4 months to NA); G12V, 16.3 months (95% CI 6.3 months to NA); Q61H, 9.5 months (95% CI 9.1 months to NA); log-rank *P* = 0.048; Figure 3E].

On multivariate analysis of OS, adjusting for age at diagnosis, performance status at registration, first-line treatment, and curative surgery status, the G12D, G12V, and Q61H mutations were identified as prognostic factors for poor OS in patients with IHC [G12D: hazard ratio (HR) 1.83, 95% CI 1.52-2.20, *P* < 0.001; G12V: HR 1.37, 95% CI 1.05-1.78, *P* = 0.019; Q61H: HR 2.03, 95% CI 1.41-2.91, *P* < 0.001; Supplementary Table S3, available at https://doi.org/10.1016/j.esmoop.2025.105306]. In patients with EHC, the G12D and G12V mutations were identified as prognostic factors for poor OS (G12D: HR 1.39, 95% CI 1.12-1.73, *P* = 0.003; G12V: HR 1.58, 95% CI 1.25-2.00, *P* < 0.001; Supplementary Table S3, available at https://doi.org/10.1016/j.esmoop.2025.105306). In patients with GB, the Q61H mutation was significantly associated with OS. However, due to the low frequency of Q61H mutations in GB patients, further accumulation of cases is needed (Supplementary Tables S1 and S3, available at https://doi.org/10.1016/j.esmoop.2025.105306).

### Association between KRAS variants and OS of BTC patients according to first-line treatment

**Figure 4:**
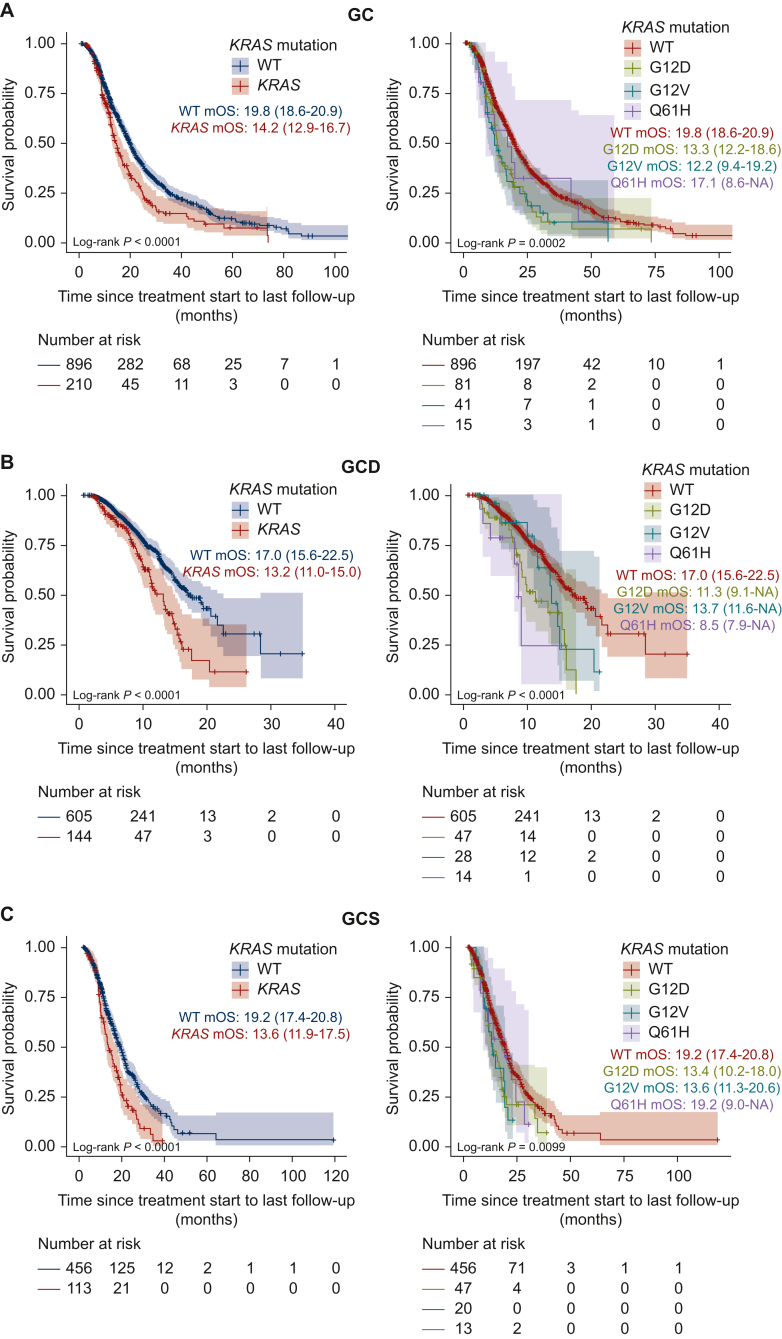
**Association between *KRAS* variants and overall survival (OS) of patients with unresectable biliary tract cancer (BTC) according to first-line treatment.** (A) Kaplan–Meier curves for unresectable BTC patients receiving GC therapy, showing OS stratified by *KRAS* variants. (B) Kaplan–Meier curves for unresectable BTC patients receiving GCD therapy, showing OS stratified by *KRAS* variants. (C) Kaplan–Meier curves for unresectable BTC patients receiving GCS therapy, showing OS stratified by *KRAS* variants. OS was measured in months from treatment initiation to last follow-up or death, and the median OS (95% CI) for each group is shown. BTC, biliary tract cancer; CI, confidence interval; GC, gemcitabine + cisplatin; GCD, gemcitabine + cisplatin + durvalumab; GCS, gemcitabine + cisplatin + S-1; mOS, median overall survival; WT, wild-type.

To investigate the impact of *KRAS* mutations on therapeutic outcomes for patients with unresectable BTC, we examined the association between *KRAS* mutation status and OS, stratified by first-line treatment. When OS was compared from the treatment initiation date, BTC patients with *KRAS* mutations had worse OS than those with WT across all treatment regimens: GC [median OS: WT, 19.8 months (95% CI 18.6-20.9 months); mutant *KRAS*, 14.2 months (95% CI 12.9-16.7 months); log-rank *P* < 0.0001; Figure 4A], GCD [median OS: WT, 17.0 months, (95% CI 15.6-22.5 months); mutant *KRAS*, 13.2 months (95% CI 11.0-15.0 months); log-rank *P* < 0.0001; Figure 4B], and GCS [median OS: WT, 19.2 months (95% CI 17.4-20.8 months); mutant *KRAS*, 13.6 months (95% CI 11.9-17.5 months); log-rank *P* < 0.0001; Figure 4C].

To further investigate the impact of the three most frequent *KRAS* mutations (G12D, G12V, and Q61H) on treatment outcomes of patients with unresectable BTC, we examined the association between *KRAS* variants and OS after each treatment regimen. *KRAS* variants were significantly associated with OS of BTC patients across all treatment regimens: GC [median OS: WT, 19.8 months (95% CI 18.6-20.9 months); G12D, 13.3 months (95% CI 12.2-18.6 months); G12V, 12.2 months (95% CI 9.4-19.2 months); Q61H, 17.1 months (95% CI 8.6 months to NA); log-rank *P* = 0.0002; Figure 4A], GCD [median OS: WT, 17.0 months (95% CI 15.6-22.5 months); G12D, 11.3 months (95% CI 9.1 months to NA); G12V, 13.7 months (95% CI 11.6 months to NA); Q61H, 8.5 months (95% CI 7.9 months to NA); log-rank *P* < 0.0001; Figure 4B], and GCS [median OS: WT, 19.2 months (95% CI 17.4-20.8 months); G12D, 13.4 months (95% CI 10.2-18.0 months); G12V, 13.6 months (95% CI 11.3-20.6 months); Q61H, 19.2 months (95% CI 9.0 months to NA); log-rank *P* = 0.0099; Figure 4C].

On multivariate analysis of OS, adjusting for age at diagnosis, performance status at registration, and tumor type, the G12D mutation was identified as a predictive factor for poor therapeutic outcomes in patients with unresectable BTC across all first-line treatment regimens (GC: HR 1.97, 95% CI 1.48-2.63, *P* < 0.001; GCD: HR 2.43, 95% CI 1.50-3.94, *P* < 0.001; GCS: HR 1.64, 95% CI 1.11-2.42, *P* = 0.013; Supplementary Table S3, available at https://doi.org/10.1016/j.esmoop.2025.105306). Additionally, the G12V mutation was identified as a predictive factor for poor therapeutic outcomes in BTC patients receiving GC therapy (HR 1.71, 95% CI 1.20-2.43, *P* = 0.003; Supplementary Table S3, available at https://doi.org/10.1016/j.esmoop.2025.105306). The Q61H mutation was also identified as a predictive factor for poor therapeutic outcomes in BTC patients receiving GCD therapy (HR 2.62, 95% CI 1.06-6.47, *P* = 0.037; Supplementary Table S3, available at https://doi.org/10.1016/j.esmoop.2025.105306).

### Genomic alterations and co-alteration patterns in BTC

**Figure 5:**
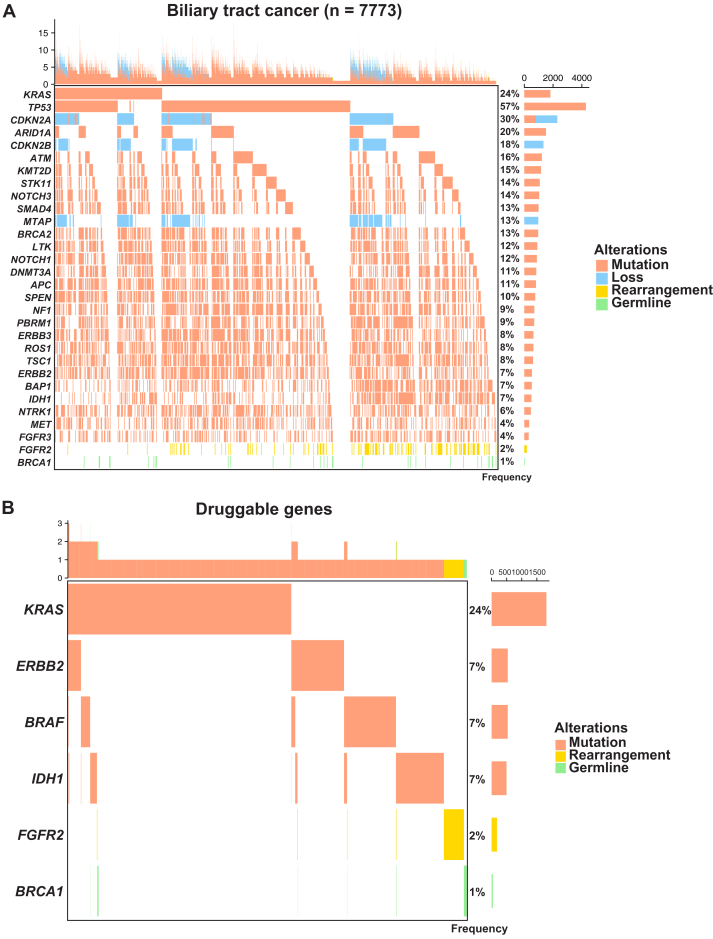
**Overall landscape of genomic alterations and co-alteration patterns in biliary tract cancer (BTC).** (A) Overview of genomic alterations, including somatic mutations, genomic losses, gene rearrangements and germline variants, and co-alteration patterns, across BTC patients. The OncoPrint displays the most frequent gene alteration events (e.g., *TP53* mutations, *CDKN2A* losses), ranked by overall frequency. Alteration types were considered separately for each gene. In addition to somatic mutations and deletions, clinically actionable events such as *FGFR2* rearrangements and germline *BRCA1* mutations were also included due to their therapeutic relevance. (B) Genomic alterations and co-alteration patterns of druggable genes (i.e. *KRAS, ERBB2, BRAF, IDH1, FGFR2,* and *BRCA1*) in BTC.

Finally, we investigated the relationship between *KRAS* mutations and other genetic alterations, as well as co-alteration patterns, in BTC. Figure 5A shows the OncoPrint illustrating the most frequent gene alteration events ranked by overall frequency in BTC. Alteration types were considered separately for each gene. In addition to somatic mutations and deletions, clinically actionable events such as *FGFR2* rearrangements and germline *BRCA1* mutations were also included due to their therapeutic relevance. This analysis revealed the overall landscape of genomic alterations and co-alteration patterns of major cancer-related genes in BTC.

We then focused on *KRAS* mutations and their co-occurrence with other alterations present in clinically druggable genes with known or potential therapeutic relevance; these included *ERBB2, BRAF, IDH1, FGFR2,* and *BRCA1*. The OncoPrint illustrates the distribution of alterations in these druggable genes across BTC patients (Figure 5B). We found that genetic alterations in these genes occurred in a mutually exclusive manner, suggesting that each may function as an independent oncogenic driver. In particular, *KRAS* mutations rarely co-occurred with other druggable gene alterations, indicating that *KRAS* variants could serve as a promising independent therapeutic target.

## Discussion

Here, we analyzed a large cohort of >7000 BTC patients and provide comprehensive evidence that *KRAS* variants have a significant effect on both survival and therapeutic outcomes after different first-line treatments in BTC patients.

The frequency of *KRAS* mutations according to tumor type differed from that reported in previous studies. We found that the overall frequency of *KRAS* mutations in BTC was 23.4%, which is higher than that in previous reports (15%-17.2%).^17^^,^^18^ After stratification by tumor subtype, we also found that the frequency of *KRAS* mutations in patients with IHC was higher in our cohort (24.9%) than in previous cohorts (7.4%-12.4%).^16^, ^17^, ^18^^,^^23^ Among patients with *KRAS* mutations, the G12D mutation was the most common in IHC (41.5%), EHC (35.9%), and GB (29.8%), which is consistent with a previous report.^17^^,^^18^ The frequency of *KRAS* variants including G12D, G12V, and Q61H in each tumor type differed from those reported previously,^17^^,^^18^ suggesting that the distribution of *KRAS* mutations differs among racial groups. Thus, the heterogeneity of *KRAS* mutations across BTC subtypes may be a target for potential personalized treatment.

Our findings reveal that BTC patients harboring *KRAS* mutations have significantly worse OS than those with WT. Recent studies also report that *KRAS* mutations are associated with poor prognosis for IHC patients^16^^,^^17^^,^^23^ and BTC patients,^18^ which is consistent with our results. This consistency across different populations reinforces the prognostic relevance of *KRAS* mutations in BTC patients and highlights their potential as a predictive biomarker for disease progression. Although previous studies report that *KRAS* mutations are associated with poor prognosis for patients with IHC,^16^^,^^17^ our study revealed that *KRAS* mutations are correlated with poor prognosis not only in IHC patients but also in EHC and GB patients.

When analyzing the association between *KRAS* variants and OS in BTC patients, we found that the G12D, G12V, and Q61H mutations were associated with poor prognosis of patients with IHC. In addition, the G12D and G12V mutations were associated with poor prognosis in patients with EHC. The association between *KRAS* G12 mutations and worse OS of IHC patients has been reported previously,^16^^,^^17^ and our findings are consistent with these results. Thus, our findings indicate that the G12D and G12V mutations are poor prognostic factors of patients with IHC and EHC. Furthermore, Q61H is a potential predictor of poor prognosis for patients with IHC. These results highlight tumor-specific differences in the prognostic relevance of *KRAS* variants.

In addition to evaluating the prognostic impact of *KRAS* mutations, we also examined their influence on the efficacy of first-line treatments for BTC (i.e. GC, GCD, and GCS). Our findings demonstrate that the *KRAS* G12D mutation was significantly associated with worse OS across all first-line treatment regimens, including GC, GCD, and GCS, indicating the strong negative impact of the G12D mutation on therapeutic outcomes in BTC patients. The G12V mutation was also associated with worse OS in BTC patients who received GC therapy. The consistent association between *KRAS* mutations and poor therapeutic outcomes highlights the urgent need to develop novel therapeutic strategies that target *KRAS*-mutated BTC. In addition, the G12D and G12V mutations were associated with poor therapeutic outcomes in BTC patients who received GCD therapy, suggesting that *KRAS* variants may predict reduced efficacy of cancer immunotherapy regimens, including ICIs. This finding is supported by research into pancreatic cancer, which shows that *KRAS* mutations create an immune-cold tumor microenvironment, potentially leading to immune evasion and resistance to ICIs.^24^

The newly developed KRAS G12C inhibitors such as sotorasib and adagrasib, which bind irreversibly to the unique cysteine residue within KRAS G12C, demonstrate promising efficacy against pancreatic cancer, non-small-cell lung cancer, and colorectal cancer.^25^, ^26^, ^27^, ^28^ An expanded phase II cohort of the KRYSTAL-1 study, designed to evaluate adagrasib in various solid tumors carrying *KRAS* G12C-mutations, treated 12 patients with advanced BTC. Partial responses were observed in 5 of the 12 patients (objective response rate, 41.7%), and disease control was achieved in nearly all patients (overall disease control rate, 91.7%).^29^ Although the frequency of *KRAS* G12C mutations in BTC was low, as reported previously,^17^^,^^18^ these findings support *KRAS* G12C as a viable therapeutic target in patients with BTC.

The most common *KRAS* variant in BTC is the G12D mutation^17^^,^^18^; therefore, much effort has been devoted to developing KRAS G12D-specific inhibitors. This approach led to development of MRTX1133, a potent and selective non-covalent inhibitor of KRAS G12D,^30^ which suppresses KRAS G12D signaling and tumor growth both *in vitro* and *in vivo*, providing a compelling rationale for clinical investigation.^30^

Beyond allele-specific inhibitors, several strategies aim to target multiple *KRAS* mutants, or the broader *RAS* family. For example, RMC-6236 is a RAS(ON) multi-selective non-covalent inhibitor that targets the active GTP-bound state of both mutant and wild-type isoforms of canonical RAS proteins.^31^ RMC-6236 shows potent anticancer activity against various *KRAS* G12X mutations (including G12D, G12V, G12R), essentially acting as a pan-KRAS inhibitor. Although BTC-specific outcomes have not yet been reported, RMC-6236 and similar agents are of potential benefit to BTC patients harboring *KRAS* mutations.

The high frequency of actionable genetic alterations in BTC highlights the importance of analyzing alterations in druggable genes to select the most appropriate treatment for each patient.^32^ Our comprehensive analysis of genomic alterations and co-alteration patterns revealed that *KRAS* mutations rarely co-occur with other druggable gene mutations such as *IDH1* mutations or *FGFR2* fusions, implying that *KRAS*-mutant BTC may represent a unique molecular subtype. Given recent advances in KRAS-targeted therapies (e.g. KRAS G12C inhibitors), this observation highlights the potential clinical utility of KRAS inhibition as a monotherapy in a distinct subset of BTC patients. The mutually exclusive distribution pattern of *KRAS* alterations further supports the rationale for precision medicine approaches.

Based on these findings, monotherapy with KRAS inhibitors, or combination therapy with KRAS inhibitors plus cancer immunotherapies such as ICIs, is expected to be highly effective in BTC patients harboring *KRAS* mutations. Future development and implementation of effective molecularly targeted therapies specific for *KRAS* variants may further expand treatment options for BTC patients.

This study has several limitations: (i) it employed a retrospective cohort design; (ii) several cases were excluded from the analysis due to incomplete prognostic or clinical data; (iii) because imaging and clinical evaluation data were not consistently available, evaluation of progression-free survival could not be performed; (iv) the duration for which GCD has been approved for BTC in Japan is relatively short; (v) our analytical approach reflects the unique medical environment in Japan, where CGP tests are covered by insurance only once and only for patients who have (nearly) completed standard treatments. Consequently, patients with rapid disease progression may miss the opportunity to undergo CGP testing.

## Conclusions

*KRAS* variants were significantly associated with poor prognosis of BTC patients. In particular, the G12D and G12V mutations were associated with poor prognosis of patients with IHC and EHC. The Q61H mutation was correlated with poor prognosis in patients with IHC. Furthermore, the G12D mutation was associated with poor therapeutic outcomes for BTC patients receiving GC, GCD, and GCS therapy. These findings indicate that *KRAS* variants may serve as potential prognostic and predictive factors for survival and therapeutic outcomes in BTC patients. In addition, since *KRAS* mutations rarely co-occur with other druggable gene mutations, *KRAS* variants could be a promising independent therapeutic target in patients with BTC.
