# Supplementary material for: Association of KRAS variants with survival and therapeutic outcomes in biliary tract cancers
Source: ESMO Open. 2025 Jun 10;10(6):105306. doi: 10.1016/j.esmoop.2025.105306 (PMC12182791; doi:10.1016/j.esmoop.2025.105306)
Supplement: Supplementary Table S1 [file mmc1.pdf]

**Supplementary Table S1. Patient Characteristics by Tumor Type**

| Characteristic | Total (n=5883) | IHC (n=2227) | EHC (n=1593) | GB (n=1388) | NA (n=675) | p-value* |
|---|---|---|---|---|---|---|
| # of patients | 5883 | 2227 | 1593 | 1388 | 675 | |
| Age at registration, years, median (IQR) | 70 [62, 75] | 70 [63, 77] | 73 [67, 78] | 70 [64, 77] | 71 [65, 77] | **<0.001** |
| Age, ≧65 (%) | 4182 (71.1%) | 1477 (66.3%) | 1253 (78.7%) | 973 (70.1%) | 479 (71.0%) | **<0.001** |
| Sex; male (%) | 3626 (61.6%) | 1386 (62.2%) | 1133 (71.1%) | 670 (48.3%) | 437 (64.7%) | **<0.001** |
| PS | | | | | | 0.08 |
| 0 (%) | 3752 (63.8%) | 1488 (66.8%) | 976 (61.3%) | 863 (62.2%) | 425 (63.0%) | |
| 1 (%) | 1921 (32.7%) | 669 (30.0%) | 557 (35.0%) | 471 (33.9%) | 224 (33.2%) | |
| 2 (%) | 129 (2.2%) | 43 (1.9%) | 34 (2.1%) | 34 (2.4%) | 18 (2.7%) | |
| 3 (%) | 22 (0.4%) | 9 (0.4%) | 4 (0.3%) | 8 (0.6%) | 1 (0.1%) | |
| 4 (%) | 5 (0.1%) | 2 (0.1%) | 2 (0.1%) | 0 (0.0%) | 1 (0.1%) | |
| Unknown (%) | 54 (0.9%) | 16 (0.7%) | 20 (1.3%) | 12 (0.9%) | 6 (0.9%) | |
| Stages of cancer before first treatment | | | | | | **<0.001** |
| I (%) | 41 (0.7%) | 13 (0.6%) | 20 (1.3%) | 5 (0.4%) | 3 (0.4%) | |
| II (%) | 210 (3.6%) | 45 (2.0%) | 118 (7.4%) | 30 (2.2%) | 17 (2.5%) | |
| III (%) | 256 (4.4%) | 78 (3.5%) | 91 (5.7%) | 75 (5.4%) | 12 (1.8%) | |
| IV (%) | 798 (13.6%) | 372 (16.7%) | 149 (9.4%) | 225 (16.2%) | 52 (7.7%) | |
| Unknown (%) | 4578 (77.8%) | 1719 (77.2%) | 1215 (76.3%) | 1053 (75.9%) | 591 (87.6%) | |
| Smoking | | | | | | **<0.001** |
| Yes (%) | 3009 (51.1%) | 1191 (53.5%) | 845 (53.0%) | 637 (45.9%) | 336 (49.8%) | |
| None (%) | 2661 (45.2%) | 955 (42.9%) | 686 (43.1%) | 704 (50.7%) | 316 (46.8%) | |
| Unknown (%) | 213 (3.6%) | 81 (3.6%) | 62 (3.9%) | 47 (3.4%) | 23 (3.4%) | |
| Alcohol polydipsia | | | | | | **<0.001** |
| Yes (%) | 896 (15.2%) | 364 (16.3%) | 241 (15.1%) | 170 (12.2%) | 121 (17.9%) | |
| None (%) | 4473 (76.0%) | 1665 (74.8%) | 1220 (76.6%) | 1109 (79.9%) | 479 (71.0%) | |
| Unknown (%) | 514 (8.7%) | 198 (8.9%) | 132 (8.3%) | 109 (7.9%) | 75 (11.1%) | |
| Metastatic status | | | | | | **<0.001** |
| Yes (%) | 5208 (88.5%) | 1970 (88.5%) | 1337 (83.9%) | 1297 (93.4%) | 604 (89.5%) | |
| None (%) | 647 (11.0%) | 245 (11.0%) | 248 (15.6%) | 86 (6.2%) | 68 (10.1%) | |
| Unknown (%) | 28 (0.5%) | 12 (0.5%) | 8 (0.5%) | 5 (0.4%) | 3 (0.4%) | |
| Resectability | | | | | | **<0.001** |
| Resectable (%) | 2757 (46.9%) | 787 (35.3%) | 986 (61.9%) | 615 (44.3%) | 369 (54.7%) | |
| Unresectable (%) | 3126 (53.1%) | 1440 (64.7%) | 607 (38.1%) | 773 (55.7%) | 306 (45.3%) | |
| CGP testing | | | | | | **<0.001** |
| FoundationOne CDx (%) | 3833 (65.2%) | 1468 (65.9%) | 1028 (64.5%) | 917 (66.1%) | 420 (62.2%) | |
| F1Liquid CDx (%) | 1160 (19.7%) | 434 (19.5%) | 349 (21.9%) | 267 (19.2%) | 110 (16.3%) | |
| NCC OncoPanel (%) | 671 (11.4%) | 253 (11.4%) | 139 (8.7%) | 143 (10.3%) | 136 (20.1%) | |
| GenMineTOP (%) | 106 (1.8%) | 39 (1.8%) | 36 (2.3%) | 27 (1.9%) | 4 (0.6%) | |
| Guardant360 CDx (%) | 113 (1.9%) | 33 (1.5%) | 41 (2.6%) | 34 (2.4%) | 5 (0.7%) | |
| Treatment group | | | | | | **<0.001** |
| GC (%) | 2079 (35.3%) | 747 (33.5%) | 581 (36.5%) | 467 (33.6%) | 284 (42.1%) | |
| GCD (%) | 1135 (19.3%) | 464 (20.8%) | 293 (18.4%) | 294 (21.2%) | 84 (12.4%) | |
| GCS (%) | 857 (14.6%) | 359 (16.1%) | 198 (12.4%) | 212 (15.3%) | 88 (13.0%) | |
| Other (%) | 1306 (22.2%) | 482 (21.6%) | 366 (23.0%) | 293 (21.1%) | 165 (24.4%) | |
| Unknown (%) | 506 (8.6%) | 175 (7.9%) | 155 (9.7%) | 122 (8.8%) | 54 (8.0%) | |
| *KRAS* mutation | | | | | | **<0.001** |
| Yes (%) | 1378 (23.4%) | 554 (24.9%) | 513 (32.2%) | 131 (9.4%) | 180 (26.7%) | |
| No (%) | 4505 (76.6%) | 1673 (75.1%) | 1080 (67.8%) | 1257 (90.6%) | 495 (73.3%) | |
| *KRAS* variant | | | | | | **<0.001** |
| G12C (%) | 67 (1.1%) | 35 (1.6%) | 20 (1.3%) | 5 (0.4%) | 7 (1.0%) | |
| G12D (%) | 531 (9.0%) | 230 (10.3%) | 184 (11.6%) | 39 (2.8%) | 78 (11.6%) | |
| G12V (%) | 315 (5.4%) | 115 (5.2%) | 137 (8.6%) | 18 (1.3%) | 45 (6.7%) | |
| G13D (%) | 93 (1.6%) | 30 (1.3%) | 30 (1.9%) | 25 (1.8%) | 8 (1.2%) | |
| Q61H (%) | 111 (1.9%) | 52 (2.3%) | 42 (2.6%) | 3 (0.2%) | 14 (2.1%) | |
| Other (%) | 261 (4.4%) | 92 (4.1%) | 100 (6.3%) | 41 (3.0%) | 28 (4.1%) | |
| WT (%) | 4505 (76.6%) | 1673 (75.1%) | 1080 (67.8%) | 1257 (90.6%) | 495 (73.3%) | |

Bold indicates $p < 0.05$.
Abbreviations: IHC, intrahepatic cholangiocarcinoma; EHC, extrahepatic cholangiocarcinoma; GB, gallbladder adenocarcinoma; NA, Not Applicable; PS, performance status; CGP, comprehensive genomic profiling.
*p-values were calculated using one-way ANOVA and Pearson's Chi-squared test.
