# Supplementary material for: Association of KRAS variants with survival and therapeutic outcomes in biliary tract cancers
Source: ESMO Open. 2025 Jun 10;10(6):105306. doi: 10.1016/j.esmoop.2025.105306 (PMC12182791; doi:10.1016/j.esmoop.2025.105306)
Supplement: Supplementary Table S2 [file mmc2.pdf]

Supplementary Table S2.
Characteristics of Patients with Unresectable BTC by First-Line Treatment

Characteristic	Total (n=2424)	GC (n=1106)	GCD (n=749)	GCS (n=569)	p-value
# of patients	2424	1106	749	569	
Age at diagnosis, years, median (IQR)	70 [62, 75]	70 [62, 75]	69 [60, 75]	67 [58, 73]	**<0.001**
Age, ≧65 (%)	1506 (62.1%)	734 (66.4%)	459 (61.3%)	313 (55.0%)	**<0.001**
Sex; male (%)	1429 (59.0%)	640 (57.9%)	429 (57.3%)	360 (63.3%)	0.055
PS					0.021
0 (%)	1514 (62.5%)	677 (61.2%)	483 (64.5%)	354 (62.2%)	
1 (%)	811 (33.5%)	386 (34.9%)	227 (30.3%)	198 (34.8%)	
2 (%)	55 (2.3%)	31 (2.8%)	19 (2.5%)	5 (0.9%)	
3 (%)	11 (0.5%)	2 (0.2%)	6 (0.8%)	3 (0.5%)	
4 (%)	2 (0.1%)	0 (0.0%)	2 (0.3%)	0 (0.0%)	
Unknown (%)	31 (1.3%)	10 (0.9%)	12 (1.6%)	9 (1.6%)	
Stages of cancer before first treatment					**<0.001**
I (%)	3 (0.1%)	1 (0.1%)	1 (0.1%)	1 (0.2%)	
II (%)	21 (0.9%)	4 (0.4%)	16 (2.1%)	1 (0.2%)	
III (%)	89 (3.7%)	24 (2.2%)	48 (6.4%)	17 (3.0%)	
IV (%)	493 (20.3%)	69 (6.2%)	373 (49.8%)	51 (9.0%)	
Unknown (%)	1818 (75.0%)	1008 (91.1%)	311 (41.5%)	499 (87.7%)	
Smoking					**0.002**
Yes (%)	1274 (52.6%)	546 (49.4%)	388 (51.8%)	340 (59.8%)	
None (%)	1065 (43.9%)	519 (46.9%)	332 (44.3%)	214 (37.6%)	
Unknown (%)	85 (3.5%)	41 (3.7%)	29 (3.9%)	15 (2.6%)	
Alcohol polydipsia					0.379
Yes (%)	388 (16.0%)	171 (15.5%)	120 (16.0%)	97 (17.0%)	
None (%)	1845 (76.1%)	859 (77.7%)	566 (75.6%)	420 (73.8%)	
Unknown (%)	191 (7.9%)	76 (6.9%)	63 (8.4%)	52 (9.1%)	
Tumor type					**<0.001**
IHC (%)	1113 (45.9%)	486 (43.9%)	362 (48.3%)	265 (46.6%)	
EHC (%)	473 (19.5%)	240 (21.7%)	132 (17.6%)	101 (17.8%)	
GB (%)	612 (25.2%)	251 (22.7%)	213 (28.4%)	148 (26.0%)	
Not Applicable (%)	226 (9.3%)	219 (11.7%)	42 (5.6%)	55 (9.7%)	
Metastatic status					0.12
Yes (%)	2076 (85.6%)	949 (85.8%)	657 (87.7%)	470 (82.6%)	
None (%)	340 (14.0%)	154 (13.9%)	90 (12.0%)	96 (16.9%)	
Unknown (%)	8 (0.3%)	3 (0.3%)	2 (0.3%)	3 (0.5%)	
CGP testing					**<0.001**
FoundationOne CDx (%)	1136 (46.9%)	521 (47.1%)	336 (44.9%)	279 (49.0%)	
F1Liquid CDx (%)	861 (35.5%)	415 (37.5%)	233 (31.1%)	213 (37.4%)	
NCC OncoPanel (%)	308 (12.7%)	145 (13.1%)	99 (13.2%)	64 (11.2%)	
GenMineTOP (%)	33 (1.4%)	6 (0.5%)	24 (3.2%)	3 (0.5%)	
Guardant360 CDx (%)	86 (3.5%)	19 (1.7%)	57 (7.6%)	10 (1.8%)	
KRAS mutation					0.912
Yes (%)	467 (19.3%)	210 (19.0%)	144 (19.2%)	113 (19.9%)	
No (%)	1957 (80.7%)	896 (81.0%)	605 (80.8%)	456 (80.1%)	
KRAS variant					0.744
G12C (%)	29 (1.2%)	15 (1.4%)	7 (0.9%)	7 (1.2%)	
G12D (%)	175 (7.2%)	81 (7.3%)	47 (6.3%)	47 (8.3%)	
G12V (%)	89 (3.7%)	41 (3.7%)	28 (3.7%)	20 (3.5%)	
G13D (%)	36 (1.5%)	15 (1.4%)	16 (2.1%)	5 (0.9%)	
Q61H (%)	42 (1.7%)	15 (1.4%)	14 (1.9%)	13 (2.3%)	
Other (%)	96 (4.0%)	43 (3.9%)	32 (4.3%)	21 (3.7%)	
WT (%)	1957 (80.7%)	896 (81.0%)	605 (80.8%)	456 (80.1%)	

Bold indicates p-value < 0.05.
Abbreviations: GC, gemcitabine + cisplatin; GCD, gemcitabine + cisplatin + durvalumab; GCS, gemcitabine + cisplatin + S-1; PS, performance status; CGP, comprehensive genomic profiling.
*p-values were calculated using one-way ANOVA and Pearson's Chi-squared test.
